# Decreased granzyme-B expression in CD11c^+^CD8^+^ T cells associated with disease progression in patients with HBV-related hepatocellular carcinoma

**DOI:** 10.3389/fimmu.2023.1107483

**Published:** 2023-01-31

**Authors:** Lin Gao, Zhixian Hong, Guanglin Lei, An-Liang Guo, Fu-Sheng Wang, Yan-Mei Jiao, Junliang Fu

**Affiliations:** ^1^ Senior Department of Infectious Diseases, The Fifth Medical Center of Chinese People's Liberation Army (PLA) General Hospital, National Clinical Research Center for Infectious Diseases, Beijing, China; ^2^ Department of Infectious Disease and Hepatology, The Second Hospital, Cheeloo College of Medicine, Shandong University, Jinan, China; ^3^ Senior Department of Hepatology, The Fifth Medical Center of Chinese People's Liberation Army (PLA) General Hospital, Beijing, China; ^4^ Center for Reproductive Medicine, Department of Obstetrics and Gynecology, Qilu Hospital of Shandong University, Jinan, China

**Keywords:** CD11c, CD8 T cells, hepatocellular carcinoma, hepatitis B virus, anti-tumor

## Abstract

**Introduction:**

CD11c^+^CD8^+^ T cells are an unconventional CD8^+^ T cell subset that exerts antiviral activity in infectious diseases. However, its characteristics in hepatocellular carcinoma (HCC) have not been elucidated.

**Methods:**

Twenty-six patients with hepatitis B virus (HBV)-related HCC and 25 healthy controls (HC) were enrolled. The frequency and phenotype of CD11c^+^CD8^+^ T cells in peripheral blood and tumors in situ were detected by flow cytometry and immunohistochemistry.

**Results:**

Both the HCC group and HC group had similar frequency and phenotype characteristics of CD11c^+^CD8^+^ T cells in the periphery. CD11c^+^CD8^+^ T cells were mainly composed of effector T cells, most of which were CD45RA^+^CCR7-. Compared with CD11c-CD8^+^ T cells, CD11c^+^CD8^+^ T cells had a higher proportion of CD38 and HLA-DR double positive, and expressed high levels of granzyme-B (GB) and degranulation marker CD107a, and produced high levels of interleukin-2 (IL-2), tumor necrosis factor alpha (TNF-α) and interferon-gamma (IFN-γ). However, the ability of degranulation and TNF-α production of CD11c^+^CD8^+^ T cells in patients with HCC were significantly lower than that in healthy controls. The GB expression level of peripheral CD11c^+^CD8^+^ T cells in patients with advanced stage of HCC was significantly lower than that in patients with early stage of HCC, and the GB expression level of liver-infiltrating CD11c^+^CD8^+^ T cells in tumor tissues was lower than that in non-tumor tissues. More importantly, the GB expression level of peripheral CD11c^+^CD8^+^ T cells was negatively correlated with tumor volume.

**Conclusions:**

These findings indicate that CD11c^+^CD8^+^ T cells may have potential anti-tumor activity and that GB^+^CD11c^+^CD8^+^ T cells are associated with disease progression in patients with HBV-related HCC.

## Introduction

Chronic hepatitis B virus (HBV) infection can lead to liver cirrhosis and liver failure and accounts for more than 80% of hepatocellular carcinoma (HCC) cases worldwide (1, 2). Viral clearance and tumor cell clearance are largely dependent on robust CD8^+^ T cell responses (3); however, T cell exhaustion occurs in various situations, leading to the loss of their ability for cytokine production or cytotoxicity against tumor cells (4–6). Moreover, cell exhaustion is also closely related to increased levels of exhaustion-related immune molecules, such as T cell Ig and ITIM domain and programmed cell death 1 (PD-1), as well as disease progression (5). Based on recent advances in CD8^+^ T cell anti-tumor therapy, some unconventional CD8^+^ T cell subsets have been identified to help reduce the tumor burden and inhibiting tumor progression (7).

CD11c is a member of the β2 integrin family, is highly expressed in dendritic cells, and is normally expressed in some B lymphocytes, CD8^+^ T cell subsets, and natural killer cells (8, 9). Previous studies have found that CD11c plays an important regulatory role in the proliferation and function of CD4^+^ and CD8^+^ T cells (10). Acute viral, bacterial, and parasitic infections lead to upregulation of the frequency of CD11c^+^CD8^+^ T cells, and this CD8^+^ T cell subset displayed characteristics of effector cells, including high activation and cytotoxic capacity to eliminate pathogenic microbes, whereas CD11c^-^CD8^+^ T cells did not (11–13).

The secretion of cytokines, such as interleukin-2 (IL-2), tumor necrosis factor alpha (TNF-α), interferon-gamma (IFN-γ), and cytolytic granules, such as granzyme B (GB), are the main functional attributes of CD8^+^ T cells that exhibit anti-tumor activity (14, 15). Moreover, tumor-infiltrating CD8^+^ T cells in colorectal cancer and lymphoma with CD11c upregulation reflect the tumoricidal efficacy (8, 16–18). The expression level of CD11c on CD8^+^ T cells could be a potential marker for evaluating tumoricidal cytotoxic T lymphocytes to predict the efficacy of anti-tumor immunotherapies (16). Although CD11c^+^CD8^+^ T cells may be potential therapeutic targets for tumors, their roles in patients with HCC remain unclear.

In this study, the phenotype and function of CD11c^+^CD8^+^ T cells in HCC patients and healthy controls (HCs) were evaluated, and the relationship between the number of CD11c^+^CD8^+^ T cells in both peripheral tissues and tumors *in situ* and HCC disease progression was further explored.

## Materials and methods

### Study participants

Twenty-six HCC patients and 25 HCs from the Fifth Medical Center of the Chinese PLA General Hospital were included in this study (detailed information of the participants was shown in **Table 1**). Peripheral blood samples were obtained from all participants, and tumor and non-tumor tissues (relatively normal liver tissue surrounding the tumor with no obvious tumor tissue observed on macroscopic observation) were obtained from nine HCC patients. Patients with concurrent hepatitis C virus and human immunodeficiency virus infections, and autoimmune or alcoholic liver disease were excluded from the study. Informed consent was obtained from all the participants in accordance with the Declaration of Helsinki. This study was approved by the Institutional Review Board of the Fifth Medical Center of the Chinese PLA General Hospital (KY-2022-4-16-1).

**Table 1:** Clinical characteristics of the enrolled patients at baseline.

Variables	HCC (n=26)	HC (n=25)	*P*
Age (year)	45 (39–52.5)	33 (31–43)	<0.01
Sex (men/women)	22/4	15/10	0.049
ALT (U/L)	29 (18–43.75)	23 (15–30)	0.058
AST (U/L)	34 (24–51.75)	23 (16–29)	0.001
TBIL (umol/L)	12.65 (9.92–19.6)	12.9 (10.5–16.4)	0.558
AFP (ng/mL)	7.35 (2.25–508.4)	–	–
HBsAg (log_10_ IU/mL)	3.58 (2.07–3.78)	–	–
HBeAg (S/Co)	0.1 (0.08–0.2)	–	–
HBV DNA (log_10_ copies/mL)	1.6 (1.59–2.1)	–	–
HCC stages (Ia/Ib/IIa/IIb/IIIa)	4/2/6/5/9	–	–

HCC, hepatocellular carcinoma; HC, health control; ALT, alanine aminotransferase; AST, aspartate aminotransferase; TBIL, total bilirubin; AFP, alpha fetoprotein; HBsAg, hepatitis B virus surface antigen; HBeAg, hepatitis B virus e antigen; HBV, hepatitis B virus. Variables were defined as non-normally distributed variables based on the results of the Kolmogorov–Smirnov test, and results are expressed as the median and interquartile range (IQR). P values were generated using the Mann-Whitney U-test (for quantitative variables) and χ2 test (for classified variables).

### Sample processing and storage

Peripheral blood mononuclear cells were isolated using Ficoll-Paque PLUS (GE Healthcare, Piscataway, NJ, USA) density gradient centrifugation and used directly in flow cytometry experiments. Freshly excised tumor tissue was fixed in 4% paraformaldehyde, embedded in paraffin, and stored in a refrigerator at -20°C.

### Flow cytometry

Peripheral blood mononuclear cells or mononuclear cells isolated from liver tissue were stained with the following antibodies for phenotypic staining: CD3-APC/Cy7, CD8-BV510, CD8-Percp, CD11c-FITC, CD11c-PE, PD-1-APC, HLA-DR-BV421, CCR7-FITC, and CD45RA-BV510. After permeabilization using a Cytofix/Cytoperm Kit (BD Biosciences, Franklin Lakes, NJ, USA), cells were used for intracellular cytokine staining using an anti-GB-PE antibody. Anti-IL-2, anti-IFN-γ, and anti-TNF-α antibodies were used to stain intracellular cytokines after stimulation with ionomycin (1 μM) and phorbol-12-myristate-13-acetate (PMA) (100 ng/mL) for 6~8 h at 37°C in the presence of 5% CO_2_. The anti-CD107a antibody was added at the same time as the stimulation. The flow cytometry antibodies were obtained from BioLegend (San Diego, CA, USA).

### Immunohistochemical staining

Paraffin-embedded and formalin-fixed liver tissue specimens was cut into 5 μm sections and placed on polylysinecoated slides. Then, the liver tissue sections were chosen to bake, deparaffinize, block the endogenous peroxidase activity with 0.3% H_2_O_2_ and heat-induce epitope retrieval. Following a brief wash and rinse in phosphate buffered saline, sections were stained with primary antibodies including mouse-anti-human CD11c and rabbit-anti-human CD8 (ZSGBBIO, Beijing, China) separately overnight at 4°C. Double staining was performed by using the HRP secondary antibody (ZSGB-BIO, China) for CD8 (blue color) and CD11c (brown color). The nuclei was stained with hematoxylin and the cytoplasm was stained with eosin. Images (100× and 400×) were acquired using Olympus CX31 microscope and Aperio VERSA 8 scanning system (Leica Microsystems, Wetzlar, Germany).

### Statistical analysis

Categorical variables were expressed as frequencies or proportions. Quantitative variables were expressed as medians and quartiles. Chi-square tests (for classified variables) and nonparametric tests (for quantitative variables) were used to compare the characteristics between the two groups (**Table 1**). The Wilcoxon paired test and Mann-Whitney U test were used to compare the median values between the two groups. Spearman’s correlation coefficient was used to assess the correlation between the two variables. The data were analyzed using SPSS 25.0 and GraphPad Prism 9.0 (GraphPad Software, San Diego, CA, USA). Statistical significance was defined as *P* < 0.05.

## Results

### Characteristics of peripheral CD11c^+^CD8^+^ T cells

**Figure 1:**
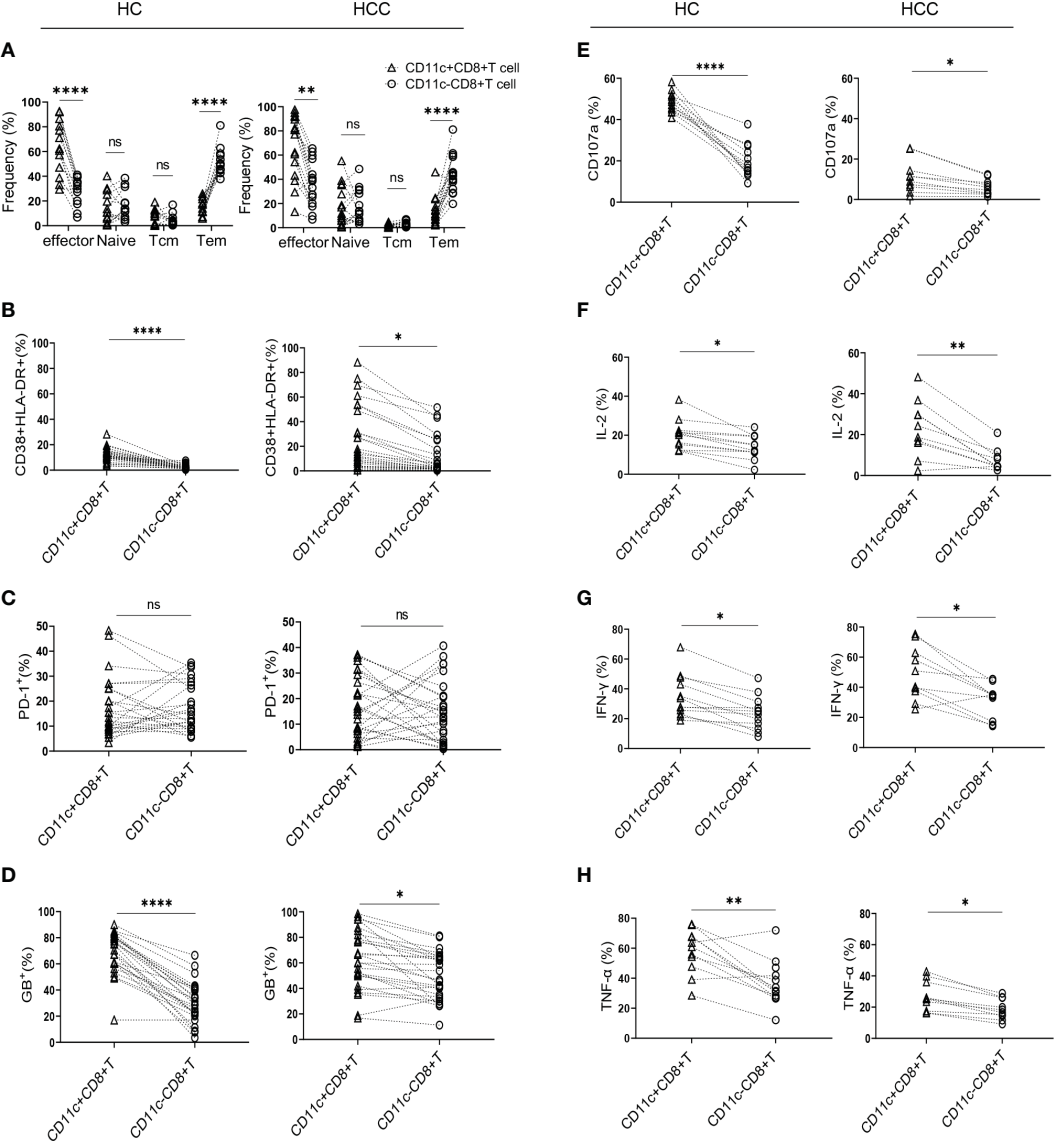
Characteristics of CD11c^+^CD8^+^ T cells in HCC and HC groups. **(A)** The frequency of effector memory (CCR7^-^CD45RA^-^) (Tem), central memory (CCR7^+^CD45RA^-^) (Tcm), naïve (CCR7^+^CD45RA^+^), and effector (CCR7^-^CD45RA^+^) subsets of CD11c^+^CD8^+^ and CD11c^-^CD8^+^ T cells in HCC (n = 16) and HC (n = 13) groups. **(B–D)** The levels of CD38&HLA-DR, PD-1, and GB expression of CD11c^+^CD8^+^ and CD11c^-^CD8^+^ T cells in HCC (n = 26) and HC (n = 25) groups. **(E–H)** After PMA stimulation, the level of CD107a expression and IL-2, IFN-γ, and TNF-α production of CD11c^+^CD8^+^ and CD11c^-^CD8^+^ T cells in HCC (n = 10) and HC (n = 12) groups. *P* values were generated using the Wilcoxon paired test between the two groups. *P < 0.05; **P < 0.01; ****P < 0.001; ns, not significant.

To determine the differentiation characteristics of CD11c^+^CD8^+^ T cells, CD45RA and CCR7 were stained in the CD8^+^ T cell populations. In both the HCC and HC groups, CD11c^+^CD8^+^ T cells mainly showed an effector phenotype (CD45RA^+^CCR7^-^), while CD11c^-^CD8^+^ T cells were mostly effector memory phenotypes (**Figure 1A**). We further analyzed the activation and exhaustion phenotype, intracellular cytokine expression, cytolytic granule GB expression, and degranulation marker CD107a in CD11c^+^CD8^+^ and CD11c^-^CD8^+^ T cells. Compared with CD11c^-^CD8^+^ T cells, CD11c^+^CD8^+^ T cells exhibited a highly activated and cytotoxic phenotype, with upregulated expression of CD38&HLA-DR, GB, and CD107a (**Figures 1B, D, E**). There were no significant differences in PD-1 expression between CD11c^+^CD8^+^ and CD11c^-^CD8^+^ T cells (**Figure 1C**). We also found that CD11c^+^CD8^+^ T cells produced higher levels of IL-2, IFN-γ, and TNF-α after PMA stimulation than the CD11c^-^CD8^+^ T cells did (**Figures 1F–H**). Representative flow cytometry plots of CD11c^-^CD8^+^ and CD11c^+^CD8^+^ T cells were shown in **Supplementary Figure 1**.

### Comparison of CD11c^+^CD8^+^ T cells between the HCC and HC groups

**Figure 2:**
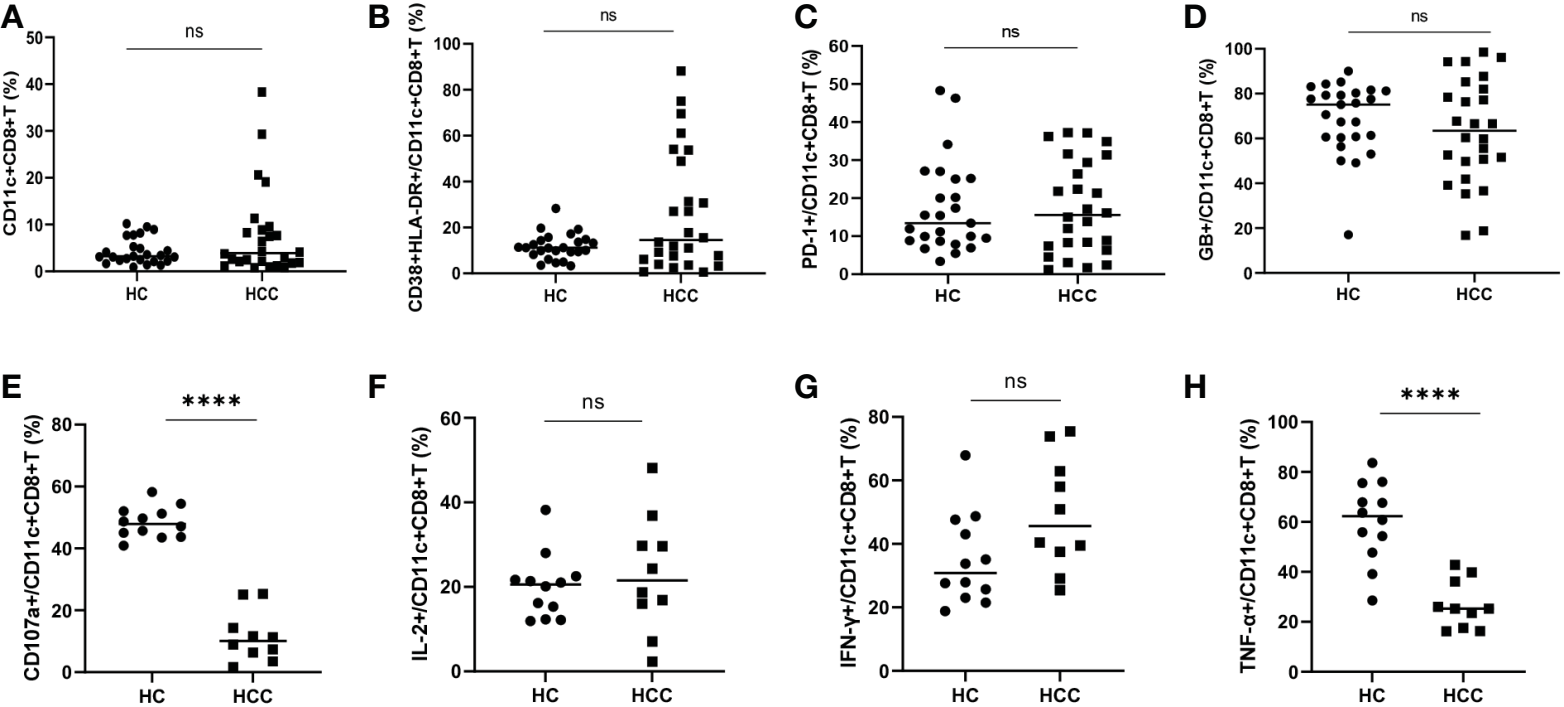
Frequency of CD11c^+^CD8^+^ T cells in HCC and HC groups. **(A)** The frequency of CD11c^+^CD8^+^ T cells in HCC (n = 26) and HC (n = 25) groups. **(B–D)** The levels of CD38&HLA-DR, PD-1, and GB expression of CD11c^+^CD8^+^ T cells in HCC (n = 26) and HC (n = 25) groups. **(E–H)** After PMA stimulation, the level of CD107a expression and IL-2, IFN-γ, and TNF-α production of CD11c^+^CD8^+^ T cells were detected in HCC (n = 10) and HC (n = 12) groups. The Mann-Whitney U test was used to compare the median values between the two groups. **** *P* < 0.001; ns, not significant.

There was no significant difference in the frequency of CD11c^+^CD8^+^ T cells and the levels of CD38&HLA-DR, PD-1, and GB expression of CD11c^+^CD8^+^ T cells between the HCC and HC groups (**Figures 2A–D**). We then compared the degranulation and cytokines production of CD11c^+^CD8^+^ T cells between the two groups. We found that the CD107a and TNF-α produced by CD11c^+^CD8^+^ T cells in HCC group were significantly decreased compared with that in HC group, while IL-2 and IFN-γ levels between the groups were comparable (**Figures 2E–H**).

### The distribution characteristics of CD11c^+^CD8^+^ T cells in tumor tissues

**Figure 3:**
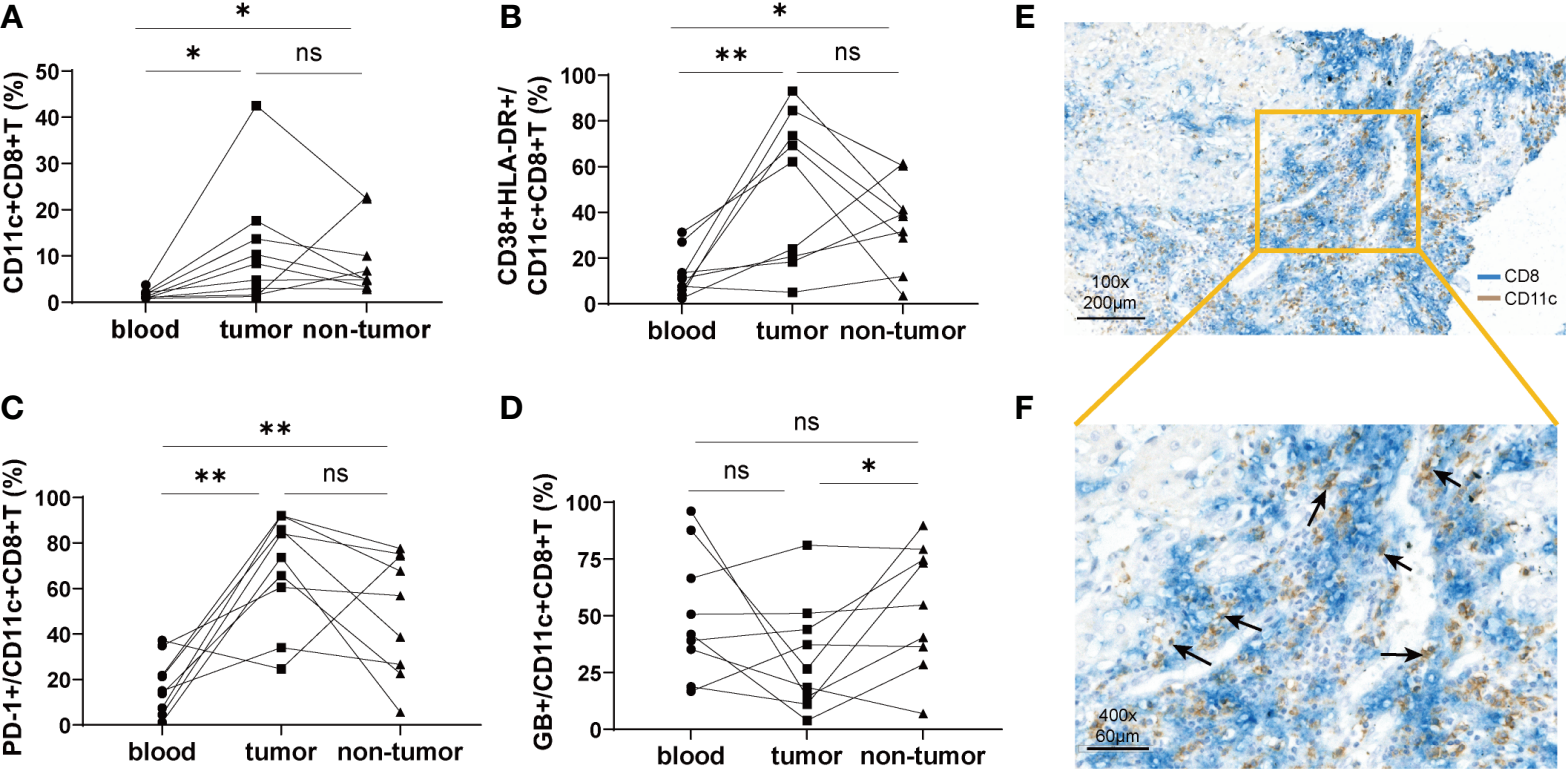
The distribution of CD11c^+^CD8^+^ T cells in tumor tissue and paired non-tumor tissue. **(A)** The frequency of CD11c^+^CD8^+^ T cells in the peripheral blood, tumor, and non-tumor tissues. **(B–D)** Paired analysis of the CD38&HLA-DR, PD-1, and GB expression on the CD11c^+^CD8^+^ T cells in the peripheral blood, tumor, and non-tumor tissues. **(E, F)** Representative results of immunohistochemical double staining of CD11c (brown) and CD8 (blue) from a patient with HCC. The orange square indicates the demarcation area between cancer and para-cancerous tissue. The black arrows point to the CD11c^+^CD8^+^ T cells. The data were from 9 HCC patients who have matched peripheral blood, tumor and non-tumor tissues. *P* values were generated using the Wilcoxon paired test between the two groups. *P < 0.05; **P < 0.01; ns, not significant.

To compare the characteristics of CD11c^+^CD8^+^ T cells in peripheral blood and liver tissue, mononuclear cells from the peripheral blood, tumor tissues, and non-tumor tissues of nine HCC patients were isolated. We found that the frequency of CD11c^+^CD8^+^ T cells in tumor and non-tumor tissues was significantly higher than that in the peripheral blood, and the difference between tumor and non-tumor tissues was not significant (**Figure 3A**). Notably, CD11c^+^CD8^+^ T cells in tumor tissues had increased expression of CD38&HLA-DR, and PD-1, and decreased expression of GB, compared with those in peripheral blood and non-tumor tissues (**Figures 3B–D**). These data indicate that CD11c^+^CD8^+^ T cells in tumor *in situ* may be in a state of high activation, exhaustion, and decreased killing capacity. The distribution of CD11c^+^CD8^+^ T cells in the liver tissue of patients with HCC was further examined by immunohistochemical staining. As shown in **Figures 3E, F**, CD11c^+^CD8^+^ T cells (black arrow) were mainly distributed at the boundary between the tumor and para-tumor tissues.

### The GB expression level of CD11c^+^CD8^+^ T cells correlates with disease progression of HCC

**Figure 4:**
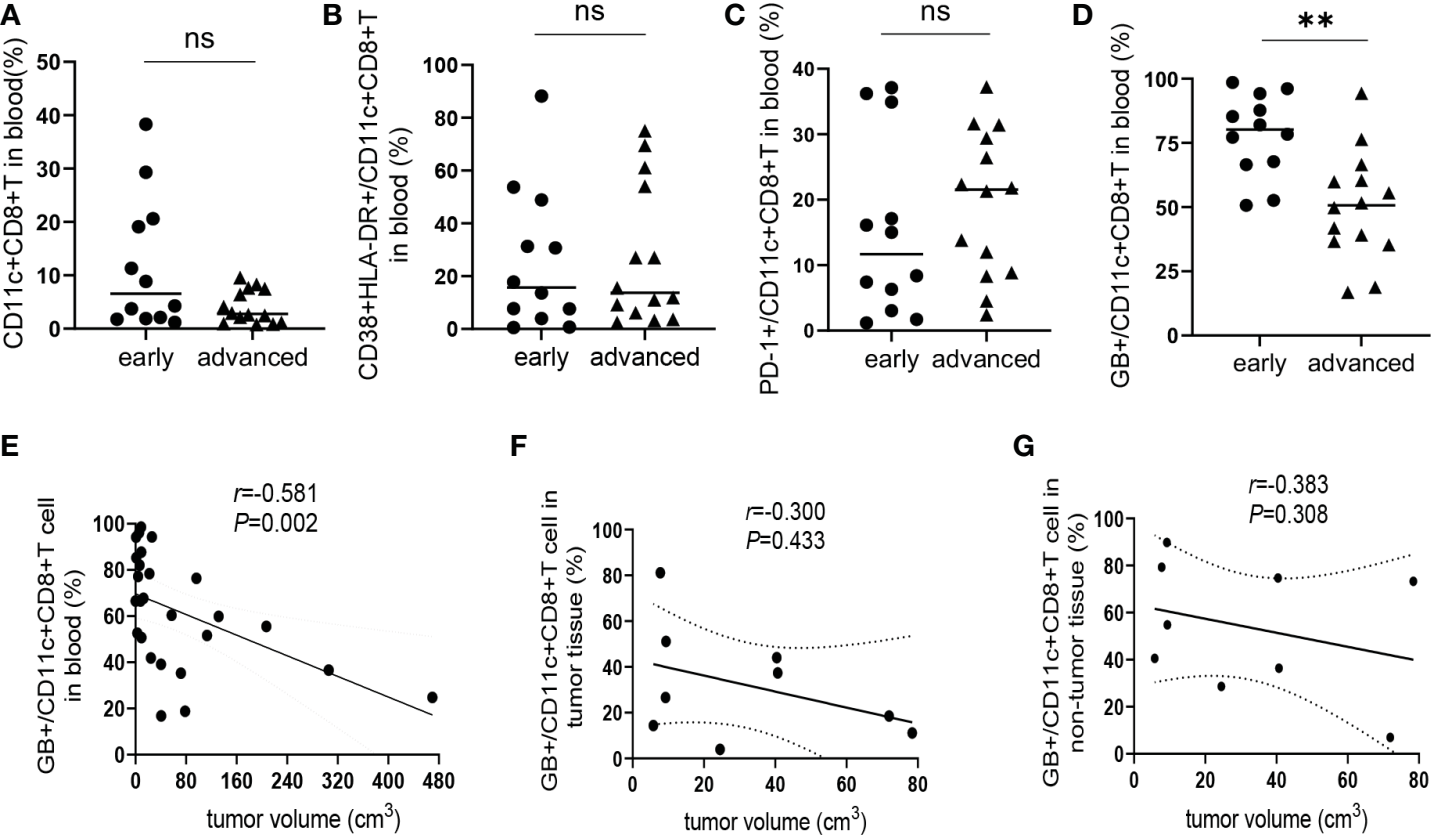
Correlation between CD11c^+^CD8^+^ T cells with estimated tumor volume. **(A)** The frequency of CD11c^+^CD8^+^ T cells in the peripheral blood of early-stage group (n = 12) and advanced-stage group (n = 14) of HCC patients. **(B–D)** The levels of CD38&HLA-DR, PD-1, and GB expression of peripheral CD11c^+^CD8^+^ T cells of early-stage group (n = 12) and advanced-stage group (n = 14) of HCC patients. **(E)** The correlation analysis between GB expression level in peripheral CD11c^+^CD8^+^ T cells and tumor volume of HCC patients (n = 26). **(F, G)** The correlation analysis between tumor volume and GB expression level in tumor and non-tumor infiltrating CD11c^+^CD8^+^ T cells of HCC patients (n = 9), respectively. The Mann-Whitney U test was used to compare the median values between the two groups. Spearman’s correlation coefficient was used to assess the correlation between the two variables. *r*, correlation coefficient. **P < 0.01; ns, not significant.

According to the standard for diagnosis and treatment of primary liver cancer (2022 edition) (19), a total of 26 HCC patients were divided into an early-stage group (stages Ia, Ib, and IIa; n = 12) and an advanced-stage group (stages IIb and IIIa; n= 14). There was no significant difference in the frequency of CD11c^+^CD8^+^ T cells and the levels of CD38&HLA-DR, and PD-1 expression on CD11c^+^CD8^+^ T cells between the early- and advanced-stage groups (**Figures 4A–C**). Meanwhile, we found that the GB expression level of CD11c^+^CD8^+^ T cells in the early-stage group was significantly higher than that in the advanced-stage group (*P* = 0.001) (**Figure 4D**). We further explored the relationship between tumor volume and the GB expression level of CD11c^+^CD8^+^ T cells in patients with HCC. Referring to a previous study, tumor volume was calculated using the equation: volume = 0.52 × width^2^ × length (20). We found that the GB expression level of CD11c^+^CD8^+^ T cells in peripheral blood was negatively correlated with tumor volume (*r* = -0.581, *P* = 0.002) (**Figure 4E**). However, there was no significant correlation between the GB expression level of CD11c^+^CD8^+^ T cells in tumor and non-tumor tissues and tumor volume (**Figures 4F, G**).

## Discussion

Tumoricidal cytotoxic T lymphocytes play an important role in viral eradication and elimination of tumors (7, 21). The effector CD8^+^ T cells are characterized by low exhaustion and high activation and proliferation (22). Previous studies have reported that CD11c^+^CD8^+^ T cells have a high proportion of antigen-specific effector cells with increased frequency following acute or chronic viral infections, such as human immunodeficiency virus type 1 or herpes simplex virus type 1 infection, and may have antiviral effects (13, 16, 23, 24). However, there are few studies on CD11c^+^CD8^+^ T cells in HBV-related HCC. In this study, we found that CD11c^+^CD8^+^ T cells of HCC patients mostly belong to effector cell subsets with higher expression levels of activated markers CD38&HLA-DR. Additionally, in both HCC and HC groups, CD11c^+^CD8^+^ T cells showed higher level of GB expression, as well as stronger ability of degranulation and IL-2, IFN-γ, and TNF-α production than CD11c^-^CD8^+^ T cells. Collectively, these findings indicate that CD11c^+^CD8^+^ T cells may be a multifunctional effector subset.

In this study, we found that the frequency of CD11c^+^CD8^+^ T cells in both tumor tissues and non-tumor tissues were significantly higher than that in peripheral blood. Combined with the data from phenotypic studies, we hypothesized that the accumulation of a large number of CD11c^+^CD8^+^ T cells in tumors is conducive to tumor control. A previous study found that CD8^+^ T cells in the tumor microenvironment have high expression levels of exhausted molecules, such as PD-1 and ITIM domains, were prone to exhaustion (25), and influence disease progression and clinical outcome (26). However, PD-1 expression is also significantly increased in activated T cells, which could be a functional effector T cell molecule, representing its active functional state (27, 28). Interestingly, our data showed that activation markers, such as CD38&HLA-DR, were highly expressed in CD11c^+^CD8^+^ T cells in tumor tissues, as well as PD-1, compared to that of CD11c^+^CD8^+^ T cells in peripheral blood. This suggests that CD11c^+^CD8^+^ T cells in the liver tumor tissues may be in a state of overactivation and exhaustion. The overactivation of immune cells usually results in increased apoptosis and decreased function. Consistent with this hypothesis, we found that the expression of GB in CD11c^+^CD8^+^ T cells in tumor tissues was significantly lower than that in non-tumor tissues.

CD107a is a marker for degranulation of intracellular cytotoxic granules. We found that the levels of CD107a expression of CD11c^+^CD8^+^ T cells after PMA stimulation in HCC group were significantly lower than that of HC group, suggested that the capacity of GB-secreting of CD11c^+^CD8^+^ T cells in HCC patients was decreased. Consistently, the capacity of TNF-α production of CD11c^+^CD8^+^ T cells was also significantly decreased in patients with HCC than in healthy controls. Most importantly, we found that the GB expression level of peripheral CD11c^+^CD8^+^ T cells in patients with advanced HCC was significantly lower than in patients with early-stage HCC. Moreover, GB^+^CD11c^+^CD8^+^ T cells in peripheral blood were negatively correlated with tumor volume. These findings suggest that the decline in the number and function of CD11c^+^CD8^+^ T cells may an important factor promoting the progression of HCC. In addition, our study is the first to observe that CD11c^+^CD8^+^ T cells were mainly distributed over the boundary of cancerous and para-cancerous tissues in the livers of patients with HCC.

This study has some limitations. First, the small sample size and lack of long-term follow-up in this cross-sectional study did not validate the role of CD11c^+^CD8^+^ T cells in predicting HCC progression. Second, there is a lack of antigen-specific tests for the number and function of CD11c^+^CD8^+^ T cells, as well as *in vivo* validation. Third, the exact mechanisms of the changes in the number and function of tumor-infiltrating CD11c^+^CD8^+^ T cells needs to be further explored.

In summary, we demonstrated that CD11c^+^CD8^+^ T cells were highly activated effector cells, and showed increased PD-1 and decreased GB expression in tumor tissues. Moreover, we found that the GB expression level of CD11c^+^CD8^+^ T cells was negatively correlated with tumor volume and associated with HCC progression. Therefore, CD11c^+^CD8^+^ T cells may be a potential prognostic marker and therapeutic target for patients with HBV-related HCC.

## Data availability statement

The original contributions presented in the study are included in the article/**Supplementary Material**. Further inquiries can be directed to the corresponding authors.

## Ethics statement

This study was approved by the institutional review board of the Fifth Medical Center of Chinese PLA General Hospital (KY-2022-4-16-1). The patients/participants provided their written informed consent to participate in this study.

## Author contributions

LG and ZH performed the literature search, conducted laboratory experiments, analyzed data, produced the figures, wrote the manuscript. GL and A-LG conducted laboratory experiments and analyzed data. F-SW assisted with data interpretation and edited the manuscript. Y-MJ and JF designed the hypothesis, analyzed data, produced the figures and wrote the manuscript. All authors contributed to the article and approved the submitted version.

## Supplementary material

Supplementary Figure 1 Gating strategy for flow cytometry analyses. Effector memory (CCR7^-^CD45RA^-^) (Tem), central memory (CCR7^+^CD45RA^-^) (Tcm), naïve (CCR7^+^CD45RA^+^), and effector (CCR7^-^CD45RA^+^) subsets, and CD38&HLA-DR, PD-1, GB expression were identified on CD11c^+^ and CD11c^-^CD8^+^CD3^+^ T cells. The production of intracellular IFN-γ, and TNF-α, and IL-2 and CD107a expression on cell membrane after PMA stimulation were identified based on CD11c^+^ and CD11c^-^CD8^+^CD3^+^ T cells. The numbers indicate the percentages of cells within the gates.
