# Supplementary figures and images for: Decreased granzyme-B expression in CD11c+CD8+ T cells associated with disease progression in patients with HBV-related hepatocellular carcinoma
Source: Front Immunol. 2023 Jan 31;14:1107483. doi: 10.3389/fimmu.2023.1107483 (PMC9927008; doi:10.3389/fimmu.2023.1107483)

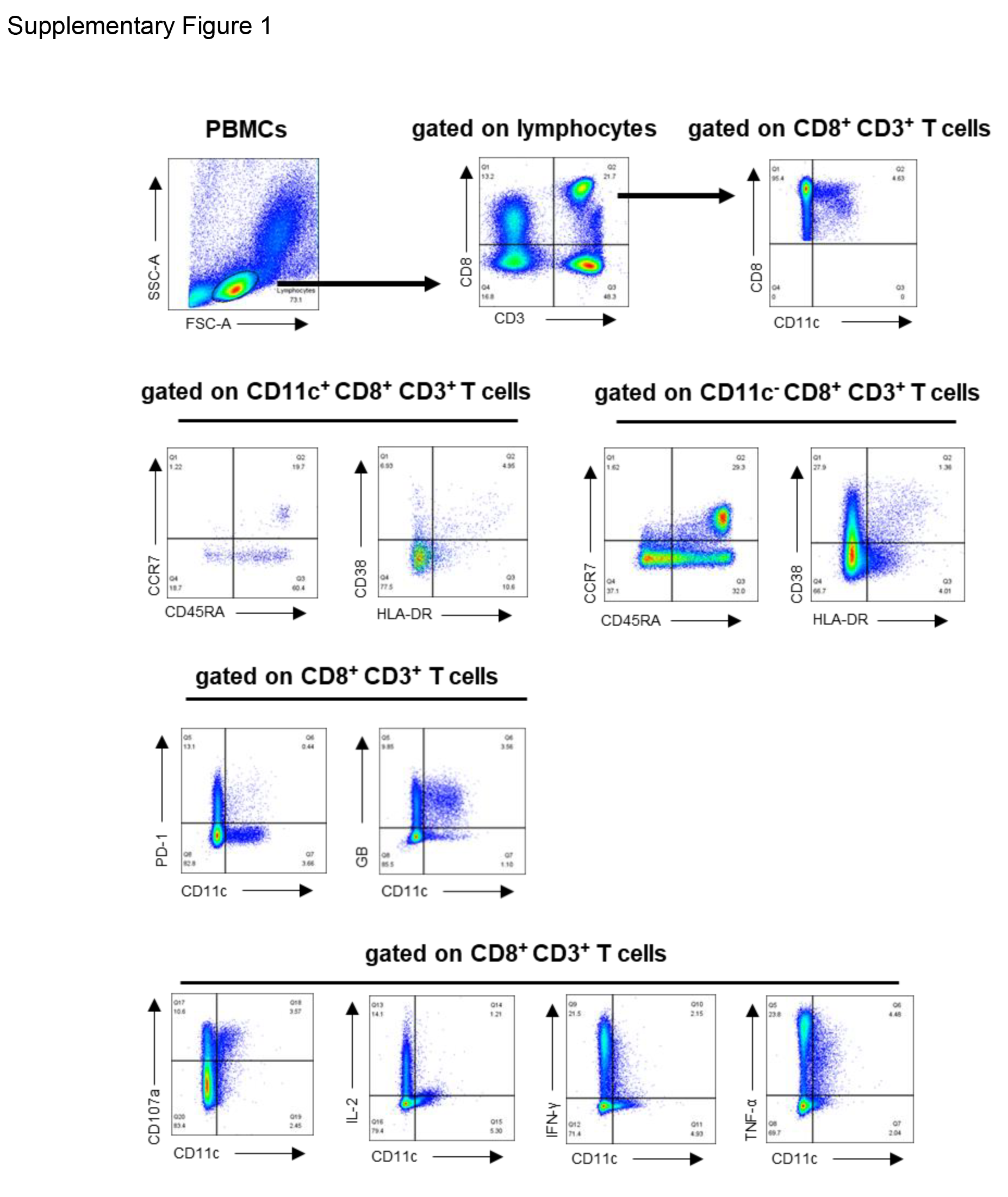

Supplement: Supplementary Figure 1 — Gating strategy for flow cytometry analyses. Effector memory (CCR7-CD45RA-) (Tem), central memory (CCR7+CD45RA-) (Tcm), naïve (CCR7+CD45RA+), and effector (CCR7-CD45RA+) subsets, and CD38&HLA-DR, PD-1, GB expression were identified on CD11c+ and CD11c-CD8+CD3+ T cells. The production of intracellular IFN-γ, and TNF-α, and IL-2 and CD107a expression on cell membrane after PMA stimulation were identified based on CD11c+ and CD11c-CD8+CD3+ T cells. The numbers indicate the percentages of cells within the gates. [file Image_1.tif]
